# Are You Tired of Working amid the Pandemic? The Role of Professional Identity and Job Satisfaction against Job Burnout

**DOI:** 10.3390/ijerph17249188

**Published:** 2020-12-09

**Authors:** Huaruo Chen, Fan Liu, Liman Pang, Fei Liu, Tingting Fang, Ya Wen, Shi Chen, Zhiyao Xie, Xuehui Zhang, Yihong Zhao, Xueying Gu

**Affiliations:** 1 School of Education Science, Nanjing Normal University, Nanjing 210046, China; 190601021@njnu.edu.cn (H.C.); 190602093@njnu.edu.cn (F.L.); 190602089@njnu.edu.cn (L.P.); 190601009@njnu.edu.cn (F.L.); 170601026@njnu.edu.cn (Y.W.); 180601021@njnu.edu.cn (S.C.); 190621111@njnu.edu.cn (Z.X.); 190621119@njnu.edu.cn (X.Z.); 190621126@njnu.edu.cn (Y.Z.); 2 Center for Research and Reform in Education, Johns Hopkins University, Baltimore, MD 21286, USA; 3 School of Education Science, Huaiyin Normal University, Huaian 223000, China; 4 School of Psychology, Nanjing Normal University, Nanjing 210046, China; 192302037@njnu.edu.cn

**Keywords:** university teachers, professional identity, job satisfaction, job burnout, COVID-19

## Abstract

With the outbreak of novel coronavirus in 2019, most universities changed from traditional offline teaching to online teaching, which brought about a large amount of problems, including teachers’ physical and mental problems. Because of teaching on the computer screen for a long period of time, the teacher lacks communication and can act casually. With long-term accumulation, the problem of teachers’ job burnout has become increasingly serious. The main purpose of this study was to examine the influence of professional identity on job burnout during the period of the novel coronavirus. At the same time, this study also discussed the moderating effect of job satisfaction on professional identity and job burnout, and its relationship between job satisfaction and job burnout. During the peak period of the COVID-19 epidemic, we conducted an online survey—483 Chinese university teachers with online teaching experience completed the Teacher Professional Identity Scale, Job Satisfaction Scale, and Job Burnout Scale. The results of this study found professional identity and job satisfaction of university teachers to be significantly negative predictors of job burnout, with job satisfaction playing a moderating role between professional identity and job burnout. This study also confirmed that professional identity and job satisfaction are important factors affecting job burnout of university teachers. Therefore, this study proposed that schools should adopt more effective strategies to improve university teachers’ professional identity and job satisfaction in order to reduce the practical problems of job burnout, ensure the effectiveness of online teaching, and maintain the sustainable development during the epidemic.

## 1. Introduction

In the outbreak of novel coronavirus pneumonia in 2019, schools shifted abruptly from traditional learning settings to online educational models. This was an experience in different educational levels, not only in primary or secondary schools but also in higher education institutions [1]. In recent years, the workload of teachers has been increasing with the expansion of university enrollment [2]. Teachers are responsible for the main teaching tasks and need to adopt various effective measures to ensure that students fully understand the knowledge they teach [3]. There is a direct effect on the normal development of activities in universities among teachers’ work activities, which means university teachers play an important role in teaching and are an indispensable part of the school’s system [4]. However, the teachers’ work and its quality requirements are increasingly demanding in the face of the impact of the epidemic situation [5]. In terms of the actual work, due to the online format, teachers must always struggle with technological glitches during their videoconference sessions, which leads them to generally feel that the work task is increasingly heavy and the work pressure ever-increasing. The ability that teachers have is not enough to solve these problems, which makes them more and more dissatisfied with work. Being unable to release tension for such a long period of time, teachers can easily be led to the decline of professional identity. Over time, teachers are prone to insomnia, tension, irritability, fatigue, and other negative emotions, resulting in varying degrees of job burnout. When the degree of teachers’ job burnout is more serious, it is easier to have a poor implementation of teaching activities and affect the order [6]. In the long run, it will affect the normal operation of university teaching, especially in the current unique situation. Therefore, we believe that it is necessary to explore the role of professional identity and job satisfaction in job burnout.

In the research on the professional problems of university teachers, only a few previous studies have simultaneously measured professional identity and job burnout. However, to our knowledge, none of the previous studies were conducted during the COVID-19 epidemic [7]. This study further researches the moderating effect of job satisfaction on job burnout and the relationship between job satisfaction and job burnout among university teachers. Reichl (2014) pointed out that the high level of university teacher job burnout may be partly due to their own personality characteristics that are not conducive to teaching management and unexpected challenges in their work, resulting in a lack of experience and cynicism [8]. Heng (2020) found that job burnout was significantly positively related to supervisor support and psychological capital [9]. Yorulmaz (2017) believed that there is a direct negative relationship between teachers’ job burnout and job satisfaction [10]. At the same time, under the current environment in China, the society has given university teachers a high social status, but their professional identity is not high. Some studies show that professional identity and job satisfaction are positively correlated and significantly correlated [11]. Therefore, to deal with the current job burnout of university teachers, it is not enough to only discuss job satisfaction, as there is also the need to enhance their professional identity and psychological motivation. In order to verify the feasibility of this measure, it is necessary to study the job burnout of university teachers and determine the effect of professional identity and job satisfaction on job burnout. On the basis of this, it is not possible to put forward effective measures to ensure the effectiveness of teachers’ work and provide a basis for further prevention and management of university teachers’ job burnout.

This study investigated the impact of university teachers’ professional identity and job satisfaction on job burnout during the COVID-19 epidemic period. The results of this study not only help researchers pay attention to the problems faced by teachers in the online teaching process but also help researchers to find more methods to help teachers solve their own psychological problems such as job burnout and professional identity caused by their own factors.

## 2. Literature Review

### 2.1. Professional Identity

Professional identity is an important research field of mental health. Many researchers have made specific definitions of professional identity of different research objects [12]. For example, Brott and Myers (1999) believed that counselors’ professional identity evolves over time, being described as a cognitive frame of reference in which they perform their professional roles and responsibilities [13]. Puglia (2008) described it in more detail that professional identity is composed of three elements: the philosophy of the profession, accreditation or certification of competence, and participation in professional bodies and activities [14]. Recently, Woo and Henfield (2015) pointed out that the current popular definition of professional identity is different, and each study on professional identity is targeted at specific groups [15], such as training consultants [16,17], practitioners [18,19], and educational consultants [20]. According to the characteristics of university teachers as the research object, this study adopted Mahmoudi et al.’s (2016) approach of professional identity as the individual’s attitude and sense of commitment to a certain occupation, which is reflected in the individual’s desire and love for the continuous work of the profession [21]. Professional identity is the determinant of teachers’ motivation, satisfaction, and job commitment, which is helpful to teachers’ retention, while the lack of these factors can easily lead to teachers’ stress and burnout [22]. Therefore, teachers’ professional identity has become an extremely important variable in academic research, one that has attracted more and more attention. For example, the study of Van Der Wal et al. (2019) combined professional identity with emotional evaluation and explored the fact that professional identity tension can lead to behavioral responses [23]. Chu (2019) showed that through teacher education projects and school cooperation projects, giving teachers certain guiding ideas and practices can help them become tutors or teacher educators, enhancing their professional identity [24]. Scartezini and Monereo (2016) explored whether the indicators of teachers’ professional identity would change when teachers and students shared what happened in the classroom during a semester. The main indicators include teachers’ statements and views on their academic roles; teachers’ concept of what it means to teach, learn, and evaluate courses taught in universities; and their feelings about the relevance of their responsibilities [25].

### 2.2. Job Satisfaction

McAllister et al. (2017) proposed that job satisfaction is an individual’s emotional state due to work experience, being a happy or positive emotional state generated when evaluating work or work experience [26]. The job satisfaction of university teachers mainly refers to the overall emotional feelings and views of their work and occupation [27], which can significantly predict the retention and performance of teachers [28]. The job satisfaction of university teachers is the key factor affecting their work attitude and behavior [29]. At present, scholars in the world have accumulated a large amount of fruitful results in the research of university teachers’ job satisfaction. The research focuses on the following three aspects: the environmental factors and personal characteristics of university teachers’ job satisfaction [30,31], and the relationship between university teachers’ job satisfaction and their work attitude and behavior. For example, Lester (1987) believed that the main factors affecting teachers’ satisfaction are school management, working environment, salary, colleague relationship, work itself, safety, and other factors [32]. Lam (2011) investigated the factors that influence teachers’ job satisfaction, and proposed that school size, working hours, leadership, work achievement, and other factors will affect teachers’ job satisfaction [33]. Wong (2010) surveyed emotional intelligence and job satisfaction of 3866 primary and secondary school teachers and middle-level leaders [34]. The results showed that the level of emotional intelligence of teachers and middle-level leaders had an effect on schoolteachers’ job satisfaction. Shaukat (2018) explored the influence of gender, age, background qualification, teaching experience, and professional qualification of Pakistani special education teachers on their self-efficacy belief and job satisfaction. The results showed that the gender, age, educational background, teaching experience, and other characteristics of teachers had a significant influence on self-efficacy belief and job satisfaction, but there was no significant correlation between self-efficacy and job satisfaction [35].

### 2.3. Job Burnout

Job burnout, defined as a syndrome characterized by emotional exhaustion, cynicism, or depersonalization, as well as a state of low professional efficacy, is a common health problem in the world today [36]. In addition to the negative impact on individual physical and mental health, burnout also affects organizational commitment, turnover intention, and job performance, leading to anxiety, depression and stress, and cardiovascular disease, affecting the positive psychological quality of individuals, such as self-esteem, life satisfaction, psychological well-being, hope, and self-efficacy [37,38,39,40,41,42,43,44]. Even studies have shown that subjects with a history of childhood abuse are particularly at risk of burnout, depression, and suicide [45]. In addition, patients with burnout may frequently display affective dysregulated temperaments that are linked to both non-suicidal self-injury and suicidal behavior [46]. Some studies have found that job burnout exists widely among professionals who provide social and human services, including teachers from different teaching fields [47]. For example, Xu (2017) studied the relationship between teacher/researcher role conflict and job burnout (including emotional exhaustion, depersonalization, and reduced personal accomplishment) [2]. The results showed that the degree of job burnout of Chinese university teachers was low to medium level, and there was a partial significant correlation between role conflict and job burnout. Heng (2020) explored the relationship between teaching research conflict and job burnout, as well as the moderating role of perceived supervisor support and psychological capital in the relationship [9]. Hierarchical regression analysis was used to investigate the moderating role of psychological support and psychological support in the relationship between teaching research conflict and job burnout. Sak (2018) compared 233 preschool teachers’ job satisfaction, job burnout, and organizational cynicism levels, paying special attention to gender differences [48]. The results showed that male and female teachers had higher organizational cynicism and lower average job satisfaction. However, the size of these differences was only moderate in the case of job satisfaction, and all other effects were small.

### 2.4. Related Research

On the basis of the description of the above three variables, this study combed the relevant research in three dimensions.

#### 2.4.1. Job Satisfaction and Job Burnout

Some studies have confirmed that job satisfaction and job burnout are related, but no research has directly confirmed the causal relationship between them, which is combined with other variables. At the same time, some studies have confirmed that this relationship also exists in teachers. For example, Atmaca (2020) explored the relationship between emotion, job burnout, and job satisfaction of teachers in Turkey [49]. A questionnaire survey was conducted among 564 teachers in different disciplines. This study successfully confirmed the five-factor model of teachers’ emotional scale. Moreover, the dimensions of happiness and love are positively correlated with job satisfaction. The dimensions of love and fear significantly predicted job satisfaction, having a moderate effect. The dimensions of love, sadness, and fear had a significant predictive effect on teachers’ job burnout, with a medium effect. Molero (2019) pointed out that job burnout is closely related to personal and environmental variables, especially job satisfaction and commitment; at the same time, the relationship between job burnout and perceptual education situation, perceived teaching efficacy (individual and collective), job satisfaction, and commitment was studied [50]. The results showed that high job burnout was related to low job satisfaction and low career commitment. Meanwhile, the research proposed that it should strengthen the education system of individual and collective ability from the perspective of developing teaching autonomy, improve the effect of personal efficacy, reduce job burnout, and improve job satisfaction.

#### 2.4.2. Professional Identity and Job Satisfaction

Some studies have found that teachers’ professional identity has a significant impact on their job satisfaction, that is, the stronger the teacher’s professional identity, the higher the job satisfaction [27]. For example, Tang’s (2020) study took rural teachers in the Chinese mainland as the research object, adopting a two-level model to explore the moderating effect of teachers’ professional identity on their job satisfaction [51]. The results confirmed that long working hours, large class size, and low cognition of income status are significantly related to low job satisfaction, while teachers’ professional identity can mitigate the negative effects of working hours and income. Karousiou (2018) mainly discussed the formation and influence of teachers’ professional identity in the “hyper diversified” school environment [52]. Through 20 interviews with 11 female teachers and 9 male teachers in 10 primary schools, the researchers showed that in the ever-changing educational environment, teachers’ professional identity and its supporting structure, such as emotion, job satisfaction, professional commitment, autonomy, and self-confidence, are often challenged and negotiated. Fuller’s (2013) research from the United Kingdom showed that teachers think their status is much lower than that of other occupations [53]. Nowadays, teachers’ job satisfaction is much lower than in the past, and the problem of teacher recruitment and retention continues. Therefore, this study used 849 pieces of survey data from the Teachers’ Association of America and in-depth interviews with 31 teachers to explore the relationship between teachers’ professional identity and status. The results showed that the recognition and reward of excellent teaching achievements do contribute to teachers’ professional identity by increasing the sense of recognition, reward, and job satisfaction.

#### 2.4.3. Professional Identity, Job Burnout, and Job Satisfaction

In the literature on teachers, there is little research on the relationship between job satisfaction, professional identity, and job burnout. The only literature found in this study is the research from Lu (2019), who conducted a survey of 267 primary and secondary school teachers from western China on their professional identity, job satisfaction, and job burnout [54]. The results showed that professional identity and job satisfaction can predict job burnout, and the influence of professional identity on job burnout is partly mediated by job satisfaction. Moreover, job satisfaction can significantly regulate the relationship between professional identity and job burnout. These results showed that professional identity and job satisfaction are important indicators of job burnout.

## 3. Materials and Methods

### 3.1. Participants

The data of this study were collected in May 2020. After the online teaching of “suspension of classes and non-stop learning” was carried out for more than 3 months in China, which meant all students were unable to attend classes in schools due to this special and extraordinary period, and by using the network platform to teach, teachers were made to teach online and students were to take online courses at home. Due to this reason, we investigated the university teachers who were conducting online teaching. The interviewees had to have had at least 3 consecutive months of online teaching experience. Finally, 500 university teachers from 15 universities in China and the United States were included in the study. Two samples were from teachers who did not carry out online teaching during the epidemic period, which did not meet the scope of this study, and 15 questionnaires from the United States were not representative. Therefore, 17 invalid or not representative questionnaires were excluded. A total of 483 pieces of data were included in the final calculation, and all of them were regarding teachers from China; at the same time, all the universities where teachers are located belong to the public, which mainly refers to schools organized by state government departments, and the school funds are basically all from government financial allocations, with these universities occupying the vast majority in China. The teachers involved in this study mainly taught in the fields of pedagogy, psychology, economics, and literature. The main reason for this is that these majors were easier to carry out online during the COVID-19 epidemic period, whereas computer science or some experimental disciplines need practical courses, and the part involving online teaching is less, which is thus not representative of the aim of this study. There were 231 male teachers and 252 female teachers involved in this study. The average age was 35.68 years (SD = 1.21). The questionnaire was completed online. All participants understood the research background, purpose, and significance of the study, and all submitted written informed consent before completing the survey.

### 3.2. Ethical Consideration

This study was conducted following the Declaration of Helsinki (2002) and Measures for Ethical Review of Biomedical Research Involving Humans, Ministry of Health, China. The protocol was approved by the Ethics Committee of Nanjing Normal University.

### 3.3. Instruments

#### 3.3.1. Professional Identity Scale

Teachers’ professional identity was assessed by the method of Song (2006) [55]. The scale has 18 items and 4 dimensions: professional value, role value, sense of belonging, and professional behavior tendency. A five-point score is used, ranging from 1 (strongly disagree) to 5 (strongly agree). The higher the score, the stronger the professional identity. In this study, Cronbach’s alpha coefficient of the scale was 0.85.

#### 3.3.2. Job Burnout Scale

Teachers’ job burnout was measured by the method of Maslach (1954) [56]. The scale has 15 topics and is a self-report scale used to describe individual job burnout. It has three dimensions: emotional exhaustion, depersonalization, and low personal accomplishment. A five-point score is used, ranging from 1 (strongly disagree) to 5 (strongly agree). In this study, the Cronbach’s alpha coefficient of the scale was 0.88.

#### 3.3.3. Job Satisfaction Scale

Job satisfaction was measured by the method of Brayfield and Rothe (1954) [57]. There are 4 items in the scale, which are scored according to 7 grades, from 1 (very disagree) to 7 (strongly agree). The higher the score, the higher the job satisfaction. In this study, Cronbach’s alpha coefficient of the scale was 0.83.

### 3.4. Research Hypothesis

According to the analysis results of the above literature review, there is a certain correlation between professional identity, job satisfaction, and job burnout. Therefore, this paper proposed the following assumptions:

**Hypothesis** **1.** *Some studies have shown that an individual’s professional identity has a significant impact on the degree of job burnout. The stronger the sense of professional identity, the less likely there is to be job burnout [58]. Therefore, as the backbone of university teaching, it is necessary to further explore the impact of university teachers’ professional identity on job burnout, so as to promote the professional development of university teachers and improve the quality and efficiency of teaching management. Hypothesis 1: Professional identity can significantly negatively predict job burnout.*

**Hypothesis** **2.** *Some studies have shown that Chinese teachers are dissatisfied with their work pressure. Many teachers feel tired and have low job satisfaction, and thus choose to leave [59]. Overall job satisfaction is negatively correlated with teachers’ job burnout, and individual job satisfaction can significantly negatively predict job burnout [60,61]. Therefore, this study proposes Hypothesis 2: Job satisfaction can significantly negatively predict job burnout.*

**Figure 1:**
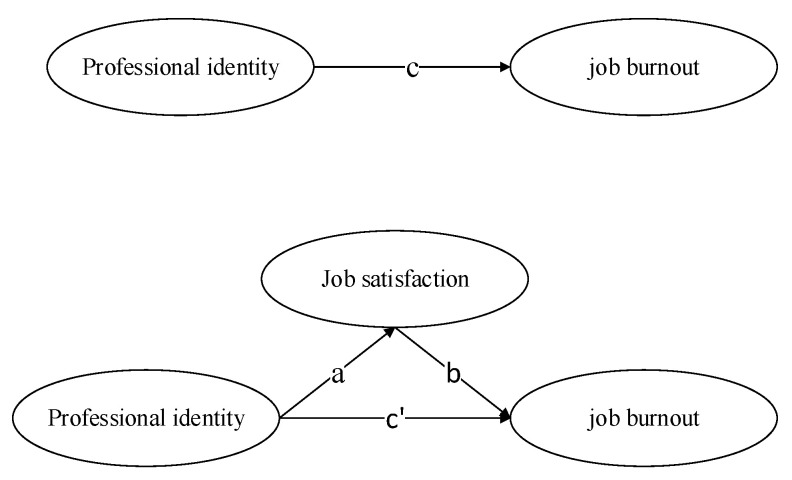
Hypothesis model.

**Hypothesis** **3.** *The research of Goulet (2002) showed that professional identity and job satisfaction are effective factors to predict job burnout. At the same time, Laurel pointed out that job satisfaction plays a role in the influence of professional identity on job burnout [62,63]. In addition, as Aryee and Tan (1992) noted, of the three dimensions, professional identity and career resilience are generally similar to Blau’s (1985) concept of career commitment [64,65]. Therefore, this study was based on the theoretical model of career commitment. In order to explore the influence of professional identity and job satisfaction on job burnout, and to verify the role that job satisfaction may play between professional identity and job burnout, this study put forward a hypothetical model. As is shown in Figure 1, a, b, and c represent direct effect, and c’ is an indirect effect.*

### 3.5. Data Processing

All data were analyzed by SPSS 25 (IBM, New York, NY, USA) and Amos 7.0 (IBM, New York, NY, USA), including common method bias test, descriptive statistics, and mediating effect analysis, etc.

## 4. Results

### 4.1. Common Method Deviation Test

Because the data of this survey were all from the self-report of university teachers, there may have been a common method deviation. Therefore, the method of Harman single factor test was used to test the deviation of variables. The results showed that the eigenvalues of 17 factors were greater than 1, and the explanatory power of the first factor was less than 40% of the critical value (the value of variation was 24.53%). Therefore, the common method bias did not affect the data results.

### 4.2. Descriptive Statistics and Correlation Analysis

According to the analysis results in Table 1, the average score of professional identity was 67.34, and the standard deviation was 8.56. The average score of job satisfaction was 21.61, and the standard deviation was 5.38. The average score of job burnout was 52.04, and the standard deviation was 2.29. According to the results of correlation analysis, there was a significant negative correlation between university teachers’ professional identity and job burnout (*p* < 0.001), a significant positive correlation with job satisfaction (*p* < 0.001), and a significant negative correlation between job satisfaction and job burnout (*p* < 0.001).

### 4.3. Mediating Effect

Hayes and Scharkow (2013) pointed out attention should be paid to the credibility of indirect effects in statistical mediation analysis, and they recommend the bias-corrected bootstrap confidence interval as the most trustworthy test [66]. Therefore, this study took professional identity as an independent variable, job burnout as a dependent variable, and job satisfaction as a mediating variable; the mediating effect of job satisfaction was analyzed by a stepwise regression method. The specific results are shown in Table 2.

It can be seen from Table 2 that the results of Equation (1) show that professional identity could significantly negatively predict job burnout (*p* < 0.001), and thus Hypothesis 1 was confirmed. The results of Equation (2) show that the independent variable professional identity could significantly positively predict the mediating variable job satisfaction (*p* < 0.001). The results of Equation (3) showed that when both professional identity and job satisfaction entered the regression equation, the mediating variable job satisfaction could significantly negatively predict job burnout (*p* < 0.001), and thus Hypothesis 2 was confirmed. At the same time, the *R*^2^ value of the independent variable professional identity to the dependent variable increased from 22.1% to 34.3%, *R*^2^ changed to 12.1%, and the regression coefficient of the independent variable to the dependent variable decreased from −0.923 to −0.704; moreover, the regression coefficient was also significant. Therefore, it can be concluded that university teachers’ professional identity can indirectly affect job burnout through the mediating role of job satisfaction.

Secondly, in Amos 21.0, the percentile and bias-corrected bootstrap CIs of Hayes and Scharkow (2013) [66] should be used to verify the mediating effect of job satisfaction. A total of 5000 samples were randomly selected from the population, and 95% bootstrap confidence interval was taken to obtain the results [66,67], as is shown in Table 3.

The results of Table 3 show that job satisfaction has a significant mediating effect on professional identity and job burnout. In the 95% confidence interval (−0.62, −0.41), 0 was not in the interval. At the same time, the direct effect of job burnout on professional identity was also significant, and 0 was also not in the interval (−0.46, −0.09). Therefore, job satisfaction was found to play a partial mediating role between university teachers’ professional identity and job burnout, and thus Hypothesis 3 was confirmed.

At the same time, this study constructed a structural equation model of job satisfaction to further verify the mediating effect of job satisfaction. In order to verify the fitting degree of the model, we used Amos 7.0 to analyze the specific fitting indexes. The results are shown in Table 4.

With reference to Schreiber et al. (2006) [68], this study compared the hypothetical model data and found that χ^2^/df = 2.663, which meets the requirements of 2 to 3; RMSEA (root mean square error of approximation) = 0.061, which meets the requirements of less than 0.06 to 0.08; NFI (normed fit index) = 0.951, IFI (incremental fit index) = 0.966, CFI (comparative fit index) = 0.957, GFI (goodness-of-fit index) = 0.956, AFI (adjusted GFI) = 0.962, which all meets the requirements of more than 0.95, indicating that the model fits well.

**Figure 2:**
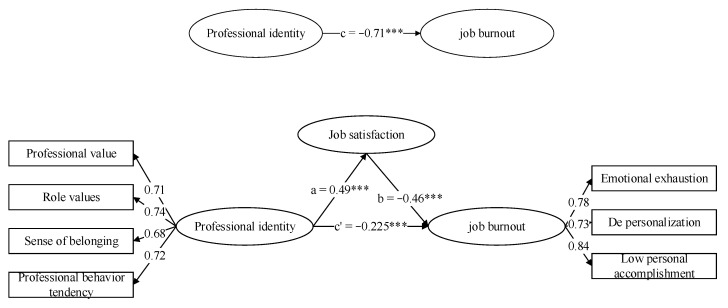
Path map of mediating role of job satisfaction. *** *p* < 0.001.

Therefore, according to the hypothetical structural equation surface model established by Hypothesis 3, this study constructed the final structural equation model. The results are shown in Figure 2.

As can be seen from Figure 2, the mediating effect of university teachers’ job satisfaction between professional identity and job burnout was 0.49 × (−0.46) = −0.225, the direct effect of job burnout on professional identity was −0.71, and the total effect of professional identity on job burnout was 0.225 + 0.71 = 0.935. The ratio of mediating effect to total effect was 24.06%.

## 5. Discussion

### 5.1. The Related Research on Professional Identity, Job Satisfaction, and Job Burnout

Consistent with the previous research results of Lu (2019) [54] in primary and secondary school teachers in China, the research results of this study confirmed that during the COVID-19 epidemic period, professional identity and job satisfaction of university teachers could also significantly negatively affect job burnout, and there was a significant positive correlation between professional identity and job satisfaction. Individuals with high professional identity had a clear understanding of their own work, accepted the nature of the work itself, adjusted their expectations appropriately, had positive professional values, and had low levels of job burnout. Individuals with high job satisfaction were more satisfied with their own work, had more positive emotions and enthusiasm at work, and had a lower level of job burnout. The results support the view that higher professional identity and job satisfaction can reduce job burnout.

### 5.2. The Mediating Effect of Job Satisfaction

The results of this study strengthen the important role of job satisfaction in reducing university teachers’ job burnout. The results show that university teachers’ professional identity not only directly affected job burnout but also indirectly affected job burnout through job satisfaction. On the one hand, professional identity was found to have a positive impact on job satisfaction. Teachers with strong professional identity had higher job satisfaction, which can reduce the possibility of job burnout. On the other hand, job satisfaction can enhance university teachers’ professional confidence and reduce job burnout. When the job satisfaction of university teachers was high, some of them showed higher professional identity. Compared with those university teachers with low professional identity and the high score of job burnout, their job burnout score was lower. Therefore, the influence of professional identity was more obvious in individuals with high job satisfaction.

### 5.3. Research Model Analysis

The results of this study confirm the hypothesis model based on Goulet’s (2002) theory of career commitment [62], that is, professional identity as an independent variable will directly affect the degree of job burnout, while job satisfaction as an intermediary will further have a negative impact on job burnout. On the basis of this, this study believes that the main reasons for obtaining this relationship are as follows: (1) according to the Social Cognitive Career Theory, the realization of human career mainly goes through three processes: belief, process, and motivation. The main relationship corresponding to this study is that professional identity is belief, the process is job satisfaction, and the motivation is job burnout. (2) As shown in this model, only when it is possible that the professional identity as a belief can be improved can job satisfaction as a process experience be ensured to be improved, and thus motivation in terms of job burnout can be effectively controlled and can then work back to the re-promotion of the professional identity itself, forming a circular system. Of course, it is inevitable that more factors will be added in this process, which will affect the whole structure model. This is also one of this study’s directions in the future.

## 6. Conclusions

The results of this study show that university teachers’ professional identity and job satisfaction can significantly negatively predict job burnout, with job satisfaction playing a partial mediating role during the COVID-19 epidemic period. The results of the study are of great significance to the development and retention of university teachers. First of all, professional identity is an important basic factor in the development of university teachers, and it is also the core of the whole teaching profession, reflecting the common needs of human beings and society. From the perspective of social culture, professional identity affects an individual’s basic work attitude, cognitions, and feelings [69]. Therefore, during the COVID-19 epidemic period, universities can change the invariable work content of university teachers through job rotation, mobilize their working enthusiasm, and make them continuously learn and improve in their work. At the same time, through a good management system, universities can promote the friendly relationship between university teachers and students, strengthen effective communication and exchange during the epidemic period, and help each other in work, all of which are also very helpful in improving the professional identity of university teachers. The third point involves the distribution of workload and time. The reason may be that university teachers are busy with repetitive and complicated work every day, especially during the COVID-19 epidemic period, with the increase of workload leading to an increase in consumption of energy and physical strength, and the enthusiasm for teaching work gradually decreasing. Therefore, universities should properly rectify the existing ways according to their own characteristics, fully reflect the self-worth of university teachers, improve the subjective initiative of university teachers, promote the unity of university teachers, and create a good humanistic environment.

This study confirms that job satisfaction plays a mediating role between teachers’ professional identity and job burnout. Therefore, teachers’ job burnout can be reduced by improving job satisfaction. As an important factor affecting teachers’ professional development, job satisfaction has become an important intervention strategy of educational psychology and vocational psychology. Thus, job satisfaction should be considered in the regular training of university teachers, even during the epidemic period—it should be considered to create more conditions to arrange the study of university teachers, establish and improve the professional learning and training system of university teachers, hold various forms of short-term training classes, organize teaching management research teams, and enhance the professional identity of university teachers through mutual learning and teaching management discussion. Regularly inviting psychological experts to carry out psychological counseling for university teachers will help release psychological pressure and will form a positive working attitude. Previous studies have shown that some newly graduated normal students will experience “reality shock” or “fail to meet expectations” when they realize that reality is not in line with their expectations after entering the teaching job [70]. Therefore, the guidance of new university teachers should be committed to making individuals more smoothly enter the role.

## 7. Limitations

There are some limitations in terms of current research. First, during the COVID-19 epidemic period, these data were only able to be obtained through online tools. Although the distribution form of this study was submitted to university teachers to fill in, which was then timely retrieved, the data basis may have had a certain deviation. Secondly, the sample size selected in this study was limited. Perhaps a larger sample size would make the effect of this study more obvious. Finally, although the results of this study confirm that there is a relationship between professional identity, job burnout, and job satisfaction, we must ask whether this result is more serious during the epidemic than before the outbreak. Because this study was not able to obtain data before the outbreak, it cannot compare and analyze the results before and after the outbreak; however, the research can explore the future after the epidemic situation is stable. This paper discusses whether the job burnout of teachers will be reduced and whether the professional identity and job satisfaction will rise when the epidemic situation is over and when offline teaching is fully resumed.

## Figures and Tables

**Table 1:** Descriptive statistics and correlation analysis matrix.

	M	SD	1	2	3
1. Professional identity	67.34	8.56	1		
2. Job satisfaction	21.61	5.38	0.68 ***	1	
3. Job burnout	52.04	2.29	−0.55 ***	−0.61 ***	1

Note: *** *p* < 0.001.

**Table 2:** Stepwise regression model of the mediating role of job satisfaction.

Regression	Dependent Variable	Predictive Variable	*R* ^2^	*β*	*T*	*F*
Equation (1)	Job burnout	Professional identity	0.221	−0.923	−7.273 ***	65.422 ***
Equation (2)	Job satisfaction	Professional identity	0.203	0.491	8.051 ***	57.668 ***
Equation (3)	Job burnout	Professional identity	0.343	−0.704	−8.712 ***	62.323 ***
		Job satisfaction		−0.462		

Note: *** *p* < 0.001.

**Table 3:** Bootstrap analysis of mediating effect significance test.

Path	Direct/Intermediate Effect Value	95% Bootstrap Confidence Interval	
		BootLLCI	BootULCI
Direct effect			
PI→JS	0.49	0.73	0.51
PI→JB	−0.71	−0.46	−0.09
JS→JB	−0.46	−0.35	−0.11
Indirect effect			
PI→JS→JB	0.49 × (−0.46) = −0.225	−0.62	−0.41

Note: PI: professional identity; JS: job satisfaction; JB: job burnout.

**Table 4:** Results of confirmatory factor analysis of the scale (*N* = 483).

χ^2^	df	χ^2^/df	RMSEA	NFI	IFI	CFI	GFI	AGFI
1217.086	457	2.663	0.061	0.951	0.966	0.957	0.956	0.962
